# Flexible docking-based molecular dynamics simulation of natural product compounds and Ebola virus Nucleocapsid (EBOV NP): a computational approach to discover new drug for combating Ebola

**DOI:** 10.1186/s12859-018-2387-8

**Published:** 2018-11-20

**Authors:** Mochammad Arfin Fardiansyah Nasution, Erwin Prasetya Toepak, Ahmad Husein Alkaff, Usman Sumo Friend Tambunan

**Affiliations:** Bioinformatics Research Group, Department of Chemistry, Faculty of Mathematics and Natural Science, Universitas Indonesia, Kampus UI, Depok, 16424 Indonesia

**Keywords:** Ebola virus, Ebola virus nucleocapsid, Natural product compounds, Virtual screening, Flexible docking, Molecular dynamics simulation

## Abstract

**Background:**

Ebola still remains as one of the most problematic infectious diseases in Africa with a high rate of mortality. Although this disease has been known for an almost half-century, there are no vaccines and drugs available in the market to treat Ebola. Zaire ebolavirus (EBOV), a single-stranded RNA virus which belongs to *Filoviridae* family and *Mononegavirales* order, is one of the virus causing Ebola. As one of seven proteins that EBOV encodes, Ebola virus nucleoprotein (EBOV NP) plays an imperative role in EBOV proliferation cycle. Therefore, the development of a new Ebola treatment can be targeted towards EBOV NP.

**Results:**

In this work, we screened about 190,084 natural product compounds from ZINC15 database through in silico virtual screening and flexible docking simulation. Furthermore, the bioavailability and toxicity prediction have been conducted as well. Two best ligands according to the simulation and prediction tests were progressed into the molecular dynamics simulation.

**Conclusion:**

In the end, we found that our proposed ligands, namely α-lipomycin (ZINC56874155) and 3-(((S)-1-amino-1,2,3,4-tetrahydroisoquinolin-5-yl)methyl)-5-((5-((5R,7S)-5,7-dihydroxy-3-oxodecyl)-2-hydroxyphenoxy) methyl)pyrrolo[3,4-b]pyrrol-5-ium (ZINC85628951), showed the promising results to be developed as a lead compounds for treating Ebola. Therefore, an experimental study is required to validate their inhibition activities against EBOV NP.

## Background

Ebola, previously known as Ebola hemorrhagic fever or Ebola virus disease is an acute viral infection with fever followed by bleeding diathesis marked by high mortality rate in human and nonhuman primates [1]. Typically, the initial infection shows no symptoms. After incubation for about 4–10 days, patient exhibits the flu-like nonspecific symptoms such as fever, myalgia, and malaise. As the infection progresses, the disease develops into severe bleeding, coagulation abnormalities, and a range of hematological irregularities. The neurological symptoms such as coma, delirium, and convulsions may also develop during the late stage of infection [2]. The patients die around 6–9 weeks after the symptoms [3]. World Health Organization (WHO) has recognized Ebola as one of the most dangerous diseases in the world due to its non-specific symptoms, severe morbidity, and high mortality rate [4].

Since it was first discovered in 1976, twenty-five Ebola outbreaks have been occurred in the world, most which mainly took place in Western and Central Africa region countries [4]. The last outbreak in 2014–2016 was the most extensive and deadliest Ebola outbreak recorded. It began in the rural area of Guinea in December 2013 and spread to urban centers of Guinea and its neighboring countries, Sierra Leone and Liberia [5]. Ebola has claimed 11,310 lives out of 28,616 reported cases when the outbreak ends in March 2016 [4, 6]. Even though the damage caused by Ebola is beyond measure, there are no FDA-approved antiviral treatments for Ebola until now. Therefore, the deployment of new antiviral drugs for Ebola is really necessary right now.

Ebola is caused by *Ebolavirus*, an enveloped, nonsegmented, negative-sense, single-stranded RNA virus which belongs to *Filoviridae* family along with *Marburgvirus* and *Cuevavirus* [7, 8]. *Ebolavirus* is subdivided into five species; Zaire ebolavirus (EBOV), Sudan ebolavirus (SUDV), Tai Forest ebolavirus (TAFV), Bundibugyo ebolavirus (BDBV), and Reston ebolavirus (RESTV) [9, 10]. The estimated case fatality rate for infection by *Ebolavirus* was 65.4% (Confidence Interval, CI, 95%). Out of five species, EBOV comes as the most devastating virus which has the highest case-fatality rate at 76% (CI 95%) [11]. On the other hand, RESTV can only infect nonhuman primates such as gorillas and chimpanzees [12, 13].

The EBOV viral genome consists of around 19,000 bases [14]. It encodes seven proteins which have an imperative role in EBOV viral life cycle, namely nucleoprotein (NP), glycoprotein (GP), RNA-dependent RNA polymerase (L), matrix protein (VP40) and three nucleocapsid proteins (VP24, VP30, and VP35) [15, 16]. The genome itself is arranged as follows: 3′-leader-NP-VP35-VP40-GP-VP30-VP24-L-trailer-5′ [14, 17].

As a negative-sense single-stranded RNA virus (-ssRNA), the RNA genome of EBOV cannot exist alone. Thus, NP must encapsidate it and further complexed with L to form ribonucleoprotein (RNP). The RNP is essential to facilitate virus replication, transcription, and assembly [18, 19]. Inside the host cell, the virion releases the RNP which serves as the template in which the L transcribes mRNAs from the RNA genome. In the late viral replication stage, the positive-strand RNA (cRNA) which complementary to the RNA genome is also produced in the form of RNP. The RNP filled with cRNA serves as the template that produces the RNP that ready to be packaged in the virion [19, 20].

Ebola virus nucleoprotein (EBOV NP) consists of 739 amino acids. Its structure can be separated into N-tail, N-lobe, C-Lobe, non-conservative region, and C-tail. [21]. This protein mediates the interaction between L and RNA genome in the virion during the transcription process [19]. NP also protects the RNA genome from degradation by exogenous nucleases or innate immune system in a host cell. As a result, NP plays a vital role for RNP to accomplish viral replication throughout the viral life cycle [22]. The EBOV -ssRNA proliferation gets disturbed with functional disorder of NP [21]. As such, EBOV NP which involves directly in the transcription, assembly, and budding of virion might become an attractive target for the antiviral development of Ebola [23, 24].

Natural products are the compounds isolated from the living organisms produced by the secondary metabolism pathways [25]. This class of compounds has been considered to be a crucial source for medicines and drugs because of their interesting bioactivities and therapeutic potential [26–28]. With the extensively available reservoir, the natural product substances can be investigated with the intention of identifying new compounds that can be either used directly as medicines or can serve as lead structures for the development of a new and more complex drug molecule, especially as new antiviral agents [29]. In addition, the natural product compounds generally have a favorable bioavailability in comparison with the synthetic drug [26]. Some successful antiviral drugs have been developed from natural product compounds, for example, zanamivir, peramivir, and lanamivir octanoate [30].

Currently, the in silico method is rapidly gaining popularity for its implementation and application in the field of medical science. This approach can leverage chemical and biological information about ligands and/or target. Most importantly, the compounds with undesirable properties can be eliminated while the most promising candidates can advance to the next analysis [31]. One way to investigate the potency of a ligand as an inhibitor of a target is molecular docking and molecular dynamics (MD) simulation. Molecular simulation estimates the ligand-target binding energy and dynamic stability by evaluating the phenomena involved in the intermolecular interaction process [32]. Cost and time of wet laboratory experiments can be drastically reduced by in silico method.

In this research, we tried to find a novel inhibitor for EBOV NP from natural product database through in silico method by employing molecular docking and MD simulation. In addition, the bioavailability and health effect prediction have also been conducted. Therefore, the potential natural product compounds that can performed as drug candidate of Ebola can be established.

## Methods

**Fig. 1:**
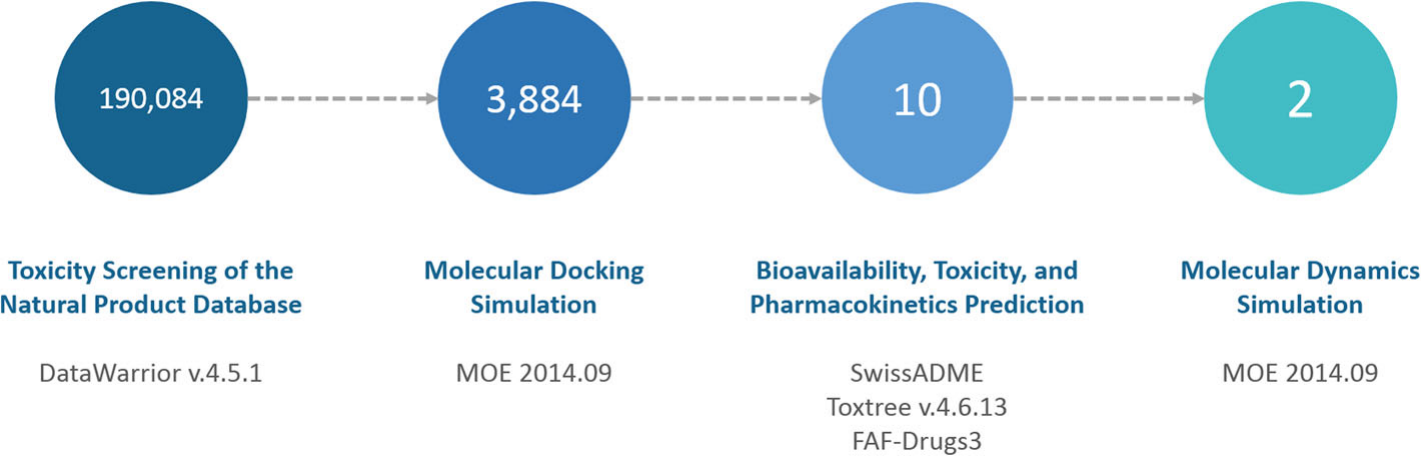
Research flowchart that was used in this study. The number inside the circles mark the number of ligands that have been used in the respective step

This research was conducted based on the validated computational approach that is developed by our research group (Fig. 1) [33, 34]. In this research, we used Personal Computer (PC) with Intel Core i7 7700 K Processor with NVidia GeForce GTX 1080 Graphics Card. We used Windows 7 Professional as Computer Operating System.

### Construction of potential natural product database

We collected about 190,084 natural product compounds from ZINC15 database [35]. To eliminate the undesirable compounds, we screened these compounds based on their toxicity properties and druglikeness score. In this research, DataWarrior v.4.5.1 software was used to predict the druglikeness and toxicity of these natural product compounds [36]. Compounds with druglikeness score below 0 and/or have either mutagenic, tumorigenic, irritant and reproductive-effect properties have been removed from the database. Furthermore, we also applied Lipinski’s Rule of Five (RO5) and Veber’s rule, with several adjustment, to reduce the number of compounds in the database [37, 38]. All of the remaining ligands were selected as potential compounds and prepared for the next step.

### Structure preparation of protein and natural product database

In this study, the structure of EBOV NP with PDB ID: 4Z9P was obtained from RCSB Protein Databank (RCSB-PDB) [21, 39]. The unwanted molecules such as water molecules in the protein structure were removed using Molecular Operating Environment 2014.09 (MOE 2014.09) software [40, 41]. We used ‘LigX’ feature on MOE to protonate and minimize the 3D structure of this protein. Parameters such as AMBER10:EHT force field, ‘Gas Phase’ solvation, and RMS gradient of 0.05 were chosen in EBOV NP structure preparation process. The “Allow ASN/GLN/HIS ‘Flips’ in Protonate3D” option was unchecked, and the default parameters were utilized on the rest.

The compounds in potential natural product databases were also prepared using the same software. These compounds were also prepared by using the default parameters in 'Wash' and 'Energy Minimization' features, with a MMFF94x force field and RMS gradient of 0.001 kcal/mol.Å were applied.

### Molecular docking simulations of natural products database

The compounds in natural product database were docked into the active site of EBOV NP using MOE 2014.09 software. The ‘Site Finder’ feature on MOE 2014.09 was used to predict the active site of EBOV NP [42]. The docking process was conducted three times. The first and second docking were performed by using ‘Rigid Receptor’ protocol. In this simulation, ‘Triangle Matcher/London dG’ and ‘Forcefield/GBVI-WSA dG’ parameters were chosen as the placement and refinement methods, respectively. Furthermore, the retain value of 30 and 100 were also selected as the retain value on placement methods in first and second docking, respectively, while the retain value of 1 was kept in both simulations on refinement method.

The third docking was carried out by using ‘Induced Fit’ protocol. In this step, the protein was made flexible to fit the conformation with the desired ligand. The rest of parameters in this docking were made with the same parameters as the previous docking simulation. At the end of the simulation, we chose the best ten ligands according to their Gibbs free binding energy (ΔG_binding_), root mean square deviation (RMSD), and binding affinity between ligands and the EBOV NP.

### Bioavailability and pharmacokinetic prediction

SwissADME (http://www.swissadme.ch/), Toxtree v2.6.13 software [43], and FAF-Drugs3 were used to predict bioavailability and pharmacokinetic properties of the best ligand from previous step result [44]. The best two out of ten ligands, based on the result of this tests, were selected to be used in the MD step.

### Molecular dynamics simulations

The stability of the EBOV NP protein complex with the best selected natural product compound was determined using MD simulations. These simulations were performed using MOE 2014.09 software. First, the selected EBOV NP-natural product compound complex was extracted from the previous simulation and saved in .moe format. This complex was then prepared using the same protocol from Section “Structure Preparation of Protein and Natural Product Database”, but instead of ‘Gas Phase’, we chose the ‘Born’ solvent as a parameter. The MD simulations were conducted by using Nosé-Poincaré-Andersen (NPA) equations in 20 ns (20.000 ps). The MD simulations have been carried out by heating the complex system from 300 K up to 312 K (temperature from the normal environment into the body temperature of Ebola patient). The simulations were ended with a cooling stage to obtain the complex structure with the lowest energy. In the end, the binding interactions of the selected compound from MD simulation were compared with the interactions from the docking simulation. Furthermore, the root mean square deviation (RMSD) values that obtained during the production stage were observed as well to determine the stability of the ligand-receptor complex during the MD simulation.

## Results and discussion

### Initial screening process

In this study, we collected about 190,084 compounds from ZINC15 database [35] and downloaded all of them in the .sdf format file. These compounds are the natural product compounds; the small compounds that are produced by living organisms [45]. Natural product compounds have frequently been used, from ancient times, to treat diseases and heal wounds [46]. Thus, natural product compounds can be potential source of antiviral drug targeting pathogenic virus, including Ebola. In this research, the initial screening process was performed to eliminate the undesirable compounds before it progressed into docking simulation process using DataWarrior v.4.5.1 software [36]. To find the molecule which has decent oral bioavailability, Lipinski’s RO5 and Veber’s rule were deployed with several exceptions. In this study, any compounds, according to Lipinski’s RO5, which has LogP lower than − 1.5 and higher than 6.6 (instead of − 0.5 and 5.6, respectively), molecular weight (MW) higher than 600 (instead of 500), hydrogen bond acceptor higher than 12 (instead of 10) and hydrogen bond donor higher than 6 (instead of 5), were eliminated. Moreover, the compounds which have rotatable bonds higher than 14 and total polar surface area (TPSA) greater than 180 Å^2^ (instead of 10 and 140 Å^2^, respectively) were also removed. Finally, any compounds with druglikeness score above 0 and do not have any mutagenic, tumorigenic, irritant and reproductive-effect properties were chosen and selected for the docking simulation.

From the initial screening process, about 63,199, 104,393, and 18,608 compounds were found to violate Lipinski’s RO5, Veber’s rule, and having either toxicity properties or lack of bioavailability score, respectively. Resulting only 3,884 compounds in the process. These compounds were selected and prepared as the ligands for the next docking simulation.

### Molecular docking simulations

Molecular docking simulation is defined as a simulation that predicts the ligand conformation and orientation (usually small molecules) in the active site of a receptor (any macromolecular target, e.g., protein or enzyme). Moreover, the docking simulation is also used to determine the ligand binding energy and free energy when it is bound with its respective binding site, creating a ligand-receptor complex, which can be computed by the software to score for selecting the best ligand [47–49]. Over the years, the docking simulation has been grown significantly and become an integral part in computer-aided drug design and development (CADDD) through virtual screening or lead-like optimization [50]. However, the rigid docking simulation may lead to false negative results (from non-bioactive compounds) and not resembling the real characteristic of the receptor, which can adapt to several conditions (e.g., temperature, and pH). Nowadays, the flexible docking, commonly known as induced-fit docking, was introduced to overcome this problem, which is more accurate and precise than rigid docking simulation, although it takes a longer time to simulate one ligand-receptor complex than the former method [51–53].

**Fig. 2:**
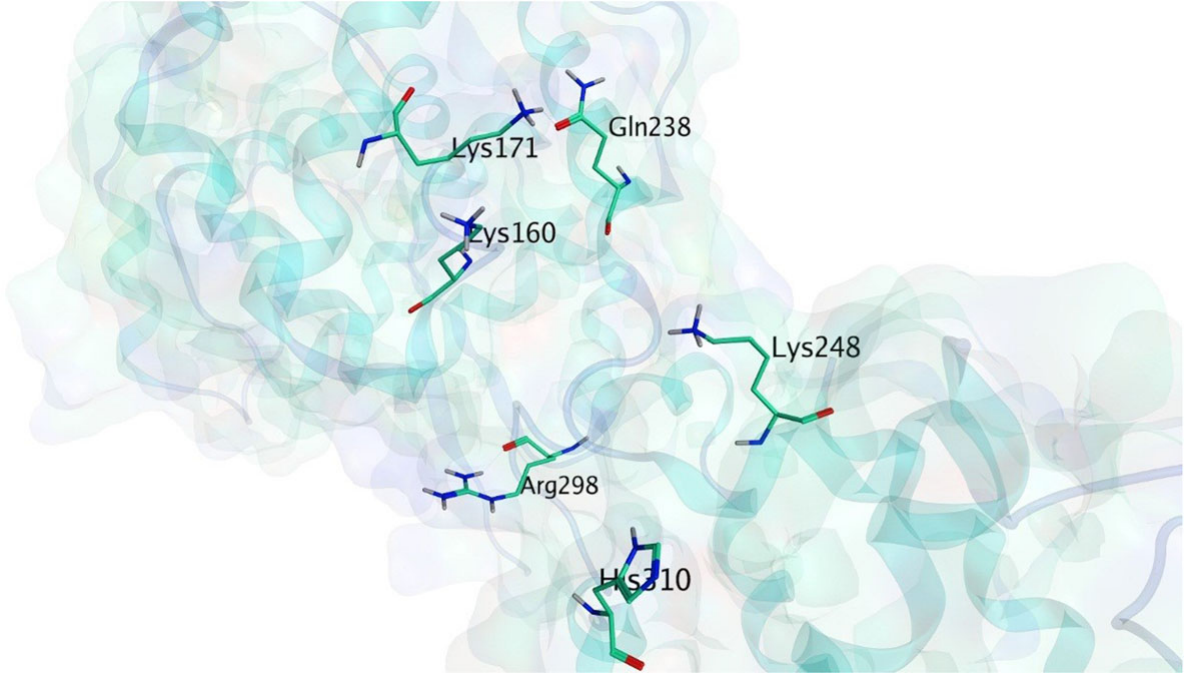
The 3D structure of Ebola nucleocapsid (NP) taken from PDB ID: 4Z9P (left), along with the binding site of EBOV NP (RNA-binding groove) according to Fu et al. in 2016

In this research, the 3D protein structure of EBOV NP (PDB code: 4Z9P) was obtained and downloaded from RCSB PDB website. Followed by the elimination of the water molecules and the addition of missing hydrogen atoms in the protein structure. Afterwards, the optimization and minimization of the 3D protein of EBOV NP using the default minimization protocol on MOE 2014.09 software were conducted. The minimization was performed with the AMBER10:EHT force field because it is suitable for protein, macromolecules and nucleic acid [54]. After the EBOV NP 3D structure was optimized, the ‘Site Finder’ feature was utilized to predict the ideal binding site of the EBOV NP. In this study, we located the EBOV NP binding site according to the recent study of Fu et al. in 2016 [42]. The result from ‘Site Finder’ feature shows that the binding site of EBOV NP comprises of twenty amino acid residues (Pro159, Lys160, Val162, Val163, Lys171, Gln238, Arg240, Phe241, Ser242, Gly243, Leu244, Leu245, Ile246, Lys248, Arg298, Val305, Asn306, Leu308, Glu309, and His310). Moreover, about six of twenty residues (Lys160, Lys171, Gln238, Lys248, Arg298, and His310) were determined as the RNA-binding groove of EBOV NP, as it can be seen in Fig. 2. Therefore, if the ligand binds perfectly with the EBOV NP on its RNA-binding groove, the interaction of EBOV NP and the viral ssRNA may be disrupted and impaired the associations of viral ssRNA [42].

In this study, about 3,884 ligands that passed the previous initial screening process were subjected into molecular docking simulation. All of these ligands were optimized and minimized through the standard protocol of ‘LigX’ feature on MOE 2014.09 software. As the standard ligands, we used licochalcone A (ZINC3873122) and 18β-glycyrrhetinic Acid (ZINC3947479) because they were previously predicted to interact with the EBOV NP at the RNA-binding site [42]. In this study, the molecular docking simulation was conducted three times; the first one was performed on 3,884 ligands, including two standard ligands, using the ‘Rigid Receptor’ protocol with the retain value of 30 [55]. In this stage, about 3,567 ligands, which have higher ΔG_binding_ value than the standards, were eliminated. Resulting only 317 ligands in the process. These ligands were further selected for the second docking simulation; using the ‘Rigid Receptor’ protocol with the retain value of 100. After the second docking simulation was conducted, we chose 100 best ligands, based on their ΔG_binding_ value, to be selected for the third docking simulation; using ‘Induced Fit’ protocol with retain value of 100. In the end, the best ten ligands that have the lowest ΔG_binding_ value of all ligands were selected, as it can be seen in Table 1.

**Table 1:** The Gibbs free binding energy (∆G_binding_), RMSD value and two-dimensional (2D) molecular structure of the ten best ligands, including two standard ligands, from docking simulation

No	ZINC Code (Molecule Name)	∆G_binding_ (RMSD)	
		Rigid Docking	Induced-fit Docking
1	ZINC14262121 (Calbistrin C)	− 7.1685 kcal/mol (3.4650)	−7.9228 kcal/mol (2.3913)
2	ZINC56874155 (α-Lipomycin)	−7.0181 kcal/mol (2.5151)	− 7.8387 kcal/mol (1.7895)
3	ZINC85596639 ((R)-4-(ethylamino)-5-(2-hydroxy-5-((2S,4S,6S)-4-hydroxy-6-(4-hydroxy-3-methoxyphenethyl)tetrahydro-2H-pyran-2-yl)-3-methoxyphenoxy)pentanoic acid)	−6.5605 kcal/mol (2.2021)	− 7.4919 kcal/mol (2.0385)
4	ZINC504747685 / ZINC218110007 (3-[(2Z)-6-hydroxy-3-oxo-2-(pyridin-3-ylmethylidene)-1-benzofuran-7-yl]-3-[3-methoxy-4-[(1-methylimidazol-2-yl)methoxy]phenyl]propanoic acid)	−7.1475 kcal/mol (1.5076)	−7.4020 kcal/mol (1.8379)
5	ZINC85628951 (3-(((S)-1-amino-1,2,3,4-tetrahydroisoquinolin-5-yl)methyl)-5-((5-((5R,7S)-5,7-dihydroxy-3-oxodecyl)-2-hydroxyphenoxy)methyl)pyrrolo[3,4-b]pyrrol-5-ium)	−7.2411 kcal/mol (1.8548)	− 7.2843 kcal/mol (1.7994)
6	ZINC85570811 (2,3-dihydroamentoflavone 7,4′-dimethyl ether)	− 6.1927 kcal/mol (2.4844)	−7.2385 kcal/mol (1.8455)
7	ZINC5431307 (Lappaol C)	− 6.5308 kcal/mol (2.4881)	− 7.2291 kcal/mol (2.0008)
8	ZINC24986227 (5-(2-(4-(3-chlorophenyl)piperazin-1-yl)ethoxy)-2-(5-methyl-4-phenyl-1H-pyrazol-3-yl)phenol)	− 6.3802 kcal/mol (2.0664)	− 7.1857 kcal/mol (2.1222)
9	ZINC85569343 ((7aS,10S,11R,11aS)-2,6,10-trihydroxy-3-(4-hydroxy-3-(3-hydroxybenzyl)-5-isobutylphenyl)-11-(hydroxymethyl)-7a,10,11,11a-tetrahydro-1H,7H-pyrano[2,3-c]xanthen-1-one)	− 6.5689 kcal/mol (1.7370)	−7.1604 kcal/mol (1.7080)
10	ZINC85837484 (Rhusflavone)	− 6.8751 kcal/mol (1.4505)	− 7.1453 kcal/mol (1.6734)
S1	ZINC3873122 (Licochalcone A)		−5.0048 kcal/mol (2.3374)
S2	ZINC3947479 (18β-Glycyrrhetinic Acid)		−5.0058 kcal/mol (3.8390)

From Table 1, calbistrin C (ZINC14262121) was chosen as the best ligand from the docking simulation because it has the lowest ΔG_binding_ value of all ligands at − 7.9228 kcal/mol, followed by α-lipomycin (ZINC56874155) as the second-lowest ΔG_binding_ value ligand at − 7.8387 kcal/mol, ZINC85596639 (− 7.4919 kcal/mol), ZINC504747685 (7.4020 kcal/mol) and ZINC85628951 (7.2843 kcal/mol). These five ligands have a ΔG_binding_ value lower than the standard ligands (− 5.0048 kcal/mol and − 5.0058 kcal/mol for the licochalcone A and 18β-glycyrrhetinic acid, respectively). This indicates that these five ligands have better inhibition potential than the standard ligands based on the ΔG_binding_. However, we must take into account that ΔG_binding_ value is not the sole factor for the inhibition potential. The RMSD value and the molecular interaction should be considered as well to determine the inhibition potential of the ligands. RMSD value from docking simulation determines the quality of ligand conformation that generated in the simulation. In this case, a binding pose of a ligand with RMSD value below 2 Å is categorized as good, acceptable binding pose. Otherwise, any ligand conformation with RMSD value above 3 Å is unacceptable [56]. For instance, we figured out from induced-fit docking simulation that calbistrin C and α-lipomycin have RMSD value of 2.3913 and 1.7895, respectively. It means that the binding pose of α-lipomycin is more acceptable than calbistrin C, even though the ΔG_binding_ value of the former is higher (more positive) than the latter.

**Fig. 3:**
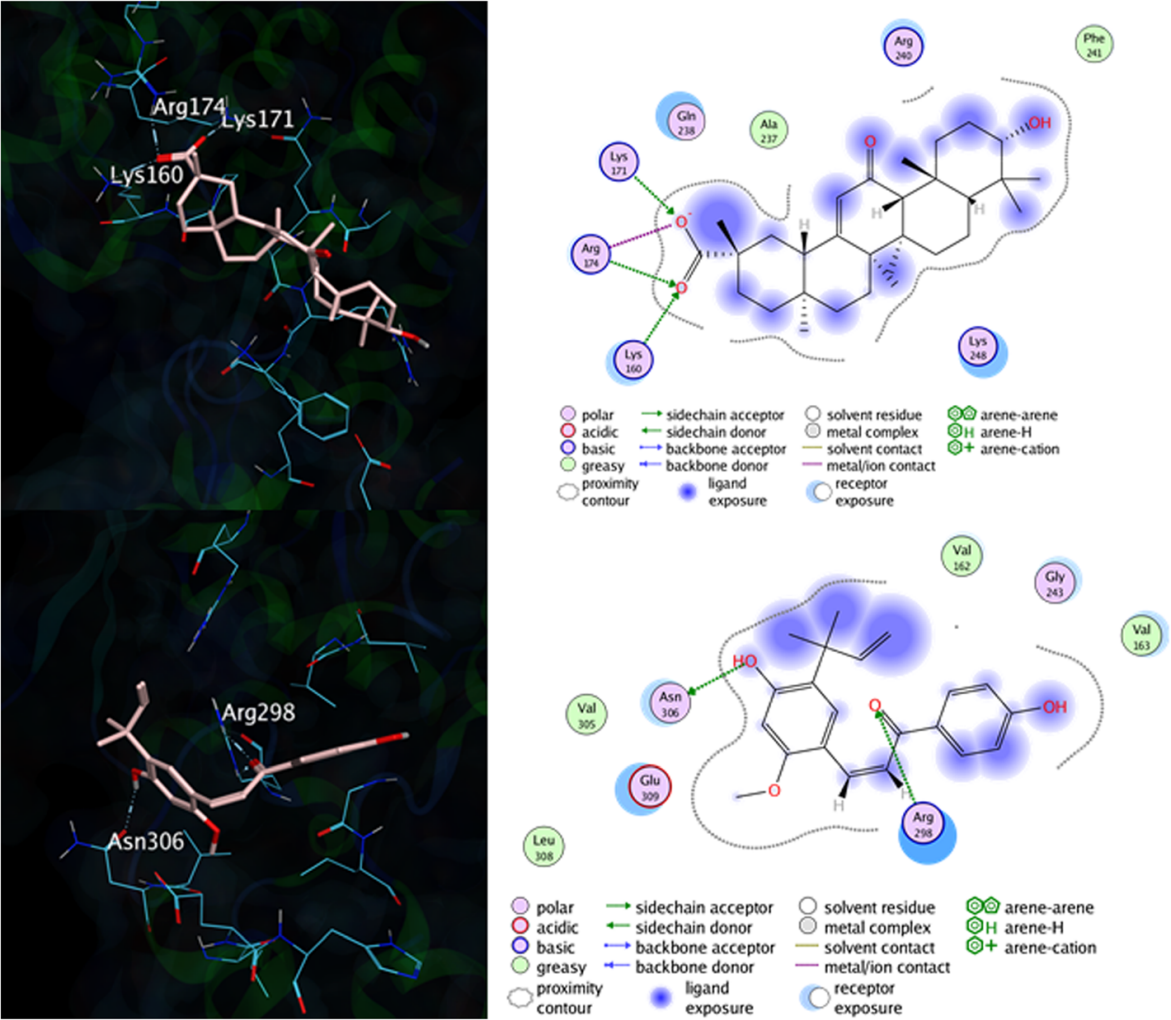
The 3D (left) and 2D (right)molecular interaction between RNA-binding groove of EBOV NP with Licochalcone A (top) and 18β-Glycyrrhetinic Acid (bottom)

The molecular interaction of the ligands in the binding site of EBOV NP can be observed by using ‘Ligand Interaction’ feature on MOE 2014.09 software, after the simulation was completed. Any interaction that happened in the simulation is considered, including the hydrogen bonds and pi-pi interactions, as well as the van der Waals interaction. For instance, the molecular interaction of the standard ligands, licochalcone A and 18β-glycyrrhetinic acid, and EBOV NP are explained in Fig. 3.

**Fig. 4:**
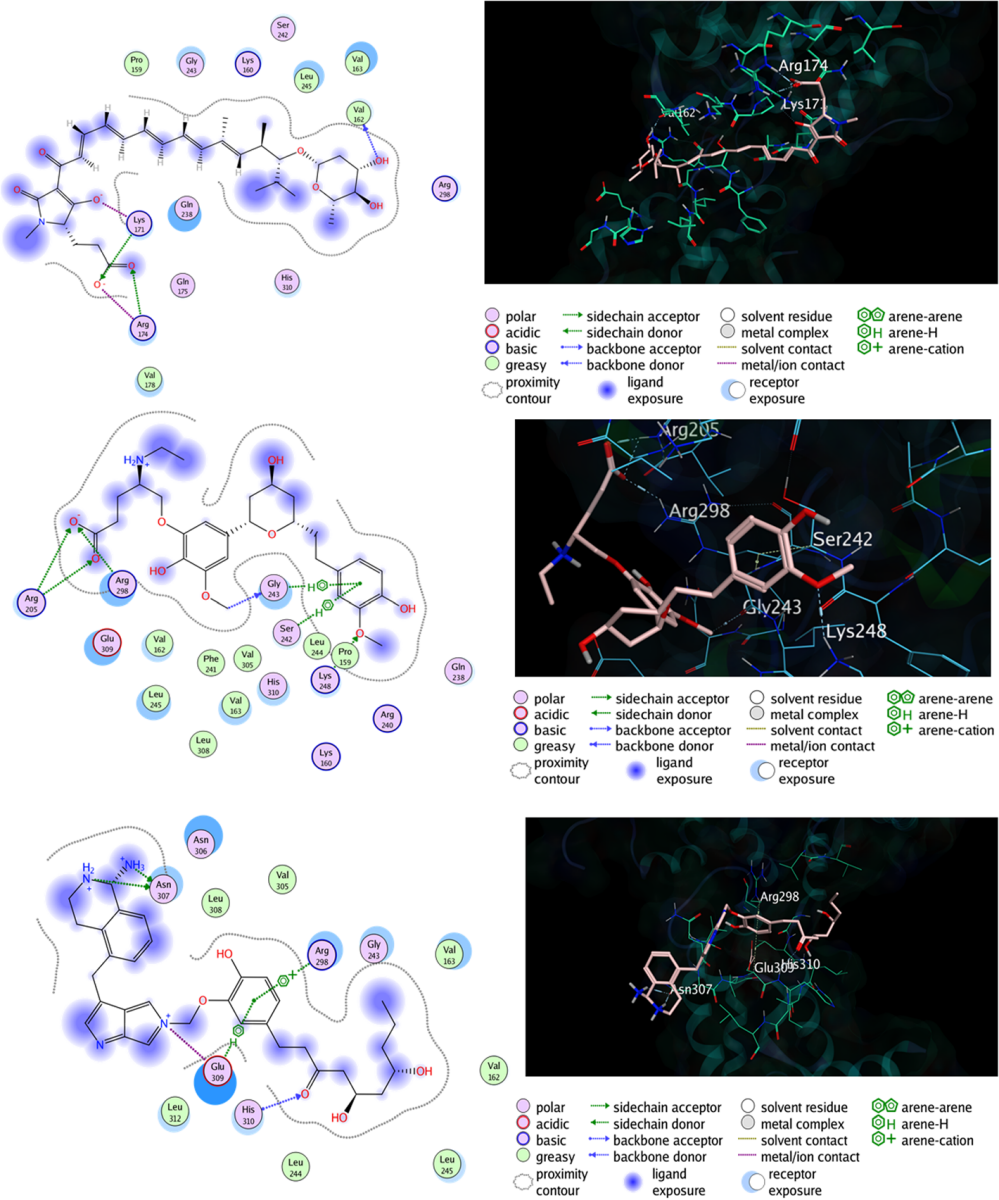
The 2D (left) and 3D (right) molecular interaction between RNA-binding groove of EBOV NP with α-lipomycin ligand (top), ZINC85596639 ligand (center), and ZINC85628951 ligand (bottom)

 It can be seen in Fig. 3, licochalcone A ligand mainly binds with the binding site of EBOV NP through two interactions; hydrogen bonds (Lys171, Lys 171, and Arg174) and van der Waals interactions (Ala237. Gln238, Arg240, Phe241, and Lys248). Meanwhile, the interaction between EBOV NP and 18β-glycyrrhetinic acid was also observed, which resulting two residues that bind through hydrogen bonds (Arg298 and Asn306) and six residues through van der Waals interaction (Val162, Val163, Gly243, Val305, Leu308, and Glu309). From the binding interaction above, we can also conclude that either licochalcone A (Lys160, Lys171, Gln238, and Lys248) and 18β-glycyrrhetinic acid (Arg298) have directly bonded with the RNA-binding groove of EBOV NP. Therefore, it is necessary to find an alternative compound that has a higher binding affinity, as well as higher Gibbs free binding energy, than these two standard ligands. Furthermore, the binding affinities of α-lipomycin was also observed, which is shown in Fig. 4.

α-lipomycin binds directly to three amino acid residues in the EBOV NP binding site, namely Val162 (-OH with the carbonyl group at the Val backbone), Lys171 (-COO^−^ and –OH with the amine group at the Lys side chain), and Arg174 (-COO^−^ with the guanidine group at the Arg side chain). Moreover, the hydrophilic area, which located in the aliphatic site of the α-lipomycin, interacts with Val163 through van der Waals interaction. Additionally, several residues, such as Pro159, Val161, Gln175, Val178, and Gln238, were also observed to interact with α-lipomycin through van der Waals interaction. Thus, α-lipomycin can be considered as good inhibitor since it blocked the Lys171 through hydrogen bond interaction, which is the one of the important residues in RNA-binding groove of EBOV NP.

The molecular interaction of ZINC85596639 and EBOV NP can be seen in Fig. 4 as well, from the figure we can observe that there are five interactions that occur in the complex, mainly pi-pi aromatic interactions (through Ser242 and Gly243) and hydrogen bond interaction (via Arg205, Gly243, and Lys248). Additionally, twelve amino acid residues were also interacted through van der Walls interaction (Pro159, Lys160, Val162, Val163, Gly238, Arg240, Phe241, Leu244, Val305, Leu308, Glu309, and His310). Thus, we confirmed that ZINC85596639 ligand might be considered as one of the potential inhibitors of EBOV NP since it can interact with four different RNA-binding groove sites through various interactions.

Finally, the molecular interaction of ZINC85628951 and EBOV NP was also observed. From the Fig. 4 we can see that six major interactions were found in the EBOV NP-ZINC85628951 complex through pi-pi interaction (Arg298 and Glu309), as well as hydrogen bond interaction (Asn307 [2×], Glu309, and His310). Furthermore, nine residues were also interacted with the ligand through van der Waals interaction. The potency of this ligand to become an inhibitor for EBOV NP is quite strong due to its interaction with Arg298 and His310, two of the RNA-binding groove site residues.

### Computational bioavailability, toxicity, and pharmacokinetics prediction

To obtain the best ligand which can be used as a lead drug for EBOV NP, the best ten ligands which previously obtained based on their Gibbs free binding energy and molecular interactions were tested for their toxicity and pharmacological properties. In order for these ligands to be functioned as a drug and can be taken orally, this ligand must pass through various tests.

In this research, the computational predictions were conducted to determine the bioavailability and pharmacokinetics, as well as physicochemical properties, of the selected ligands from the previous simulation. In this stage, we deployed ten ligands from docking simulation to be predicted by using SwissADME (http://www.swissadme.ch/) and FAF-Drugs3 [44]. As shown in Table 2, except ZINC24986227, all of the ligands have violated any of Lipinski’s RO5 or Veber’s rule, most of them by having higher MW than 500, or greater TPSA value than 140 Å^2^. The enormous MW and TPSA value of these ligands may affect the gastrointestinal (GI) absorption, as most of the ligands were predicted to have low absorption on this system. Moreover, the bioavailability score of all ligands were not impressive, as it can be seen in Table 3, with two out of ten ligands (ZINC14262121 and ZINC504747685) have the highest bioavailability score at 0.56, indicate that the bioavailability of these two ligands is slightly better than the other eight ligands. However, as shown in the table, the oral bioavailability of all ligands was considered as good, according to Egan et al. [57]. It indicates that all of the ligands may be suitable and absorbed well in our body. However, it has to be confirmed later on through in vivo studies to measure the oral bioavailability rate of these compounds.

**Table 2:** The physiochemical properties of the best ten and two standard ligands

No	ZINC ID	Physicochemical Properties				
		MW	LogP (o/w)	H-Acc	H-Bond	TPSA
1	ZINC14262121	542.66	3.67	8	4	141.36
2	ZINC56874155	587.70	3.41	9	4	153.83
3	ZINC85596639	533.61	2.23	10	5	146.94
4	ZINC504747685	527.52	2.88	9	2	133.00
5	ZINC85628951	573.70	1.95	8	5	140.41
6	ZINC85570811	568.53	4.04	10	4	155.89
7	ZINC5431307	554.58	2.90	10	5	155.14
8	ZINC24986227	489.01	4.71	4	2	64.62
9	ZINC85569343	586.63	4.03	9	6	160.82
10	ZINC85837484	540.47	3.25	10	6	177.89
S1	Licochalcone A	338.40	3.98	4	2	66.76
S2	18β-Glycyrrhetinic Acid	470.68	5.15	4	2	74.60

**Table 3:** The pharmacokinetics properties, oral bioavailability, and toxicity properties prediction of the best ten and two standard ligands

No	ZINC ID	Pharmacokinetics				MedChem	Oral Bioavailability	
		GI Absorption	Bioavailability Score	CYP Inhibitor	Solubility Index	PAINS	Veber	Egan
1	ZINC14262121	Low	0.56	CYP3A4	Good	0	Low	Good
2	ZINC56874155	Low	0.11	CYP3A4	Good	0	Low	Good
3	ZINC85596639	Low	0.55	None	Good	0	Low	Good
4	ZINC504747685	Low	0.56	CYP2C9, CYP3A4	Good	0	Good	Good
5	ZINC85628951	High	0.55	None	Good	0	Low	Good
6	ZINC85570811	Low	0.55	CYP2C9	Reduced	0	Good	Good
7	ZINC5431307	Low	0.55	CYP3A4	Reduced	0	Low	Good
8	ZINC24986227	High	0.55	CYP2C19, CYP2D6	Reduced	0	Good	Good
9	ZINC85569343	Low	0.17	CYP2C9	Reduced	0	Good	Good
10	ZINC85837484	Low	0.17	CYP2C9	Reduced	0	Good	Good
S1	Licochalcone A	High	0.55	CYP1A2, CYP2C19, CYP2D6, CYP3A4	Good	0	Good	Good
S2	18β-Glycyrrhetinic Acid	High	0.56	None	Reduced	0	Good	Good

In addition to oral bioavailability and pharmacokinetics prediction, the medicinal chemistry aspects of these ligands were also observed, which generated by FAF-Drugs3 software. In this study, we checked the pan-assay interference compounds (simply known as PAINS). Compounds that belong to PAINS have promiscuous behavior that shows apparent bioactivity. Not only that, but these compounds could also interfere the readouts from an assay. Compounds which have a substructure of PAINS are unsuitable to be lead compounds, in particular for the drug [58–60]. From Table 3, we found out that all of our ligands were not indicated to have any PAINS compounds, which means that all of the ligands are not likely to produce false-positives in high-throughput screen test [58]. Moreover, the potency of these ligands to become CYP inhibitors were also observed. This test was completed by using SwissADME software as well. In this study, all of the ligands, apart from ZINC85596639 and ZINC85628951 have the potency to become the CYP inhibitors. Surprisingly, licochalcone A ligand was predicted to inhibit at least four out from five CYP enzymes that simulated in this study, namely CYP1A2, CYP2C19, CYP2D6, CYP3A4. Therefore, this study explains that ZINC85596639 and ZINC85628951 ligands can be prepared as drug compounds without worrying that these ligands would be transformed into another compound by CYP enzymes in the human body.

The final toxicity test in this research was performed to determine the mutagenicity and carcinogenicity potential of the ligands, based on the Benigni-Bossa rule. This rule states that the mutagenic and carcinogenic potency of the ligand can be found through the fragments of the functional groups that the ligand possessed. The functional groups that have been identified as either mutagenic or carcinogenic by this rule are acyl halide, haloalkene, epoxide, aliphatic halogen, alkyl nitrate, aldehyde, hydrazine, isocyanate, polyaromatic hydrocarbon, azide, alkyl/aromatic nitro, coumarin, diazo aromatic, benzyl sulfinyl ether, alkyl halide and thiocarbonyl [61]. This prediction test was done by using Toxtree v2.6.13 software, which the results can be seen in Table 4.

**Table 4:** The mutagenicity and carcinogenicity prediction of the best ten and two standard ligands

No	ZINC ID	Negative for genotoxic carcinogenicity	Negative for nongenotoxic carcinogenicity	Potential Salmonella typhimurium TA100 mutagen based on QSAR	Potential carcinogen based on QSAR
1	ZINC14262121	Yes	No	No	No
2	ZINC56874155	No	No	No	No
3	ZINC85596639	Yes	Yes	No	No
4	ZINC504747685	Yes	Yes	No	No
5	ZINC85628951	Yes	Yes	No	No
6	ZINC85570811	Yes	No	No	No
7	ZINC5431307	Yes	Yes	No	No
8	ZINC24986227	Yes	Yes	No	No
9	ZINC85569343	Yes	Yes	No	No
10	ZINC85837484	Yes	No	No	No
S1	Licochalcone A	No	Yes	No	No
S2	18β-Glycyrrhetinic Acid	No	Yes	No	No

The parameters outlined in this test include genotoxic carcinogenicity, non-genotoxic carcinogenicity, QSAR carcinogenicity, and mutagenic potential of *Salmonella typhimurium* bacteria. Genotoxic carcinogens occur as a result of direct irreversible DNA genetic damage, whereas non-genotoxic carcinogens occur as a result of inducing cancer via other mechanisms, such as modulation of certain hormones or proteins, immune system disorders, and intercellular communication disorders, and do not directly affect DNA [61].

Based on these tests, it appears that seven out of ten ligand inhibitors have no carcinogenic or mutagenic properties, with α-lipomycin, 2,3-dihydroamentoflavone 7,4′-dimethyl ether, and rhusflavone were predicted to be a non-genotoxic carcinogenic agent. Surprisingly, α-lipomycin were also predicted as a genotoxic carcinogenic agent as well; this may happen due to the α,β unsaturated carbonyl fragment that lies in the ligand, while substituted n-alkyl carboxylic acid that also resides in α-lipomycin was the main reason why this ligand was predicted to be a non-genotoxic carcinogen agent. Furthermore, 2,3-dihydroamentoflavone 7,4′-dimethyl ether and rhusflavone were predicted as non-genotoxic carcinogenic ligands because they have o-phenylphenol fragments in their molecular structure. However, based on this test, both standard ligands were also observed and predicted to be genotoxic carcinogenic agents as well, due to the alkenyl benzene and α, β-unsaturated carbonyl fragments in the licochalcone A and 18β-glycyrrhetinic acid molecule structure, respectively.

After the computational bioavailability, toxicity, and pharmacokinetics prediction were conducted, α-lipomycin and ZINC85628951 ligands were chosen for the preparation of MD simulation, based on the results of docking simulation, as well as from bioavailability and pharmacokinetics prediction. The former ligand was chosen because it has the second-lowest of the ∆G_binding_ value of all ligands and good oral bioavailability (according to Egan, but not with Veber), although it potentially harmful to our body due to being a carcinogenic agent, while the latter ligand was selected not only because it has high GI absorption and solubility, but also predicted to be a safe compound because it is not predicted to be either carcinogenic or mutagenic agent. Moreover, ZINC85628951 ligand was also predicted to become non-CYP enzymes inhibitor as well.

### Results of molecular dynamics simulations

**Fig. 5:**
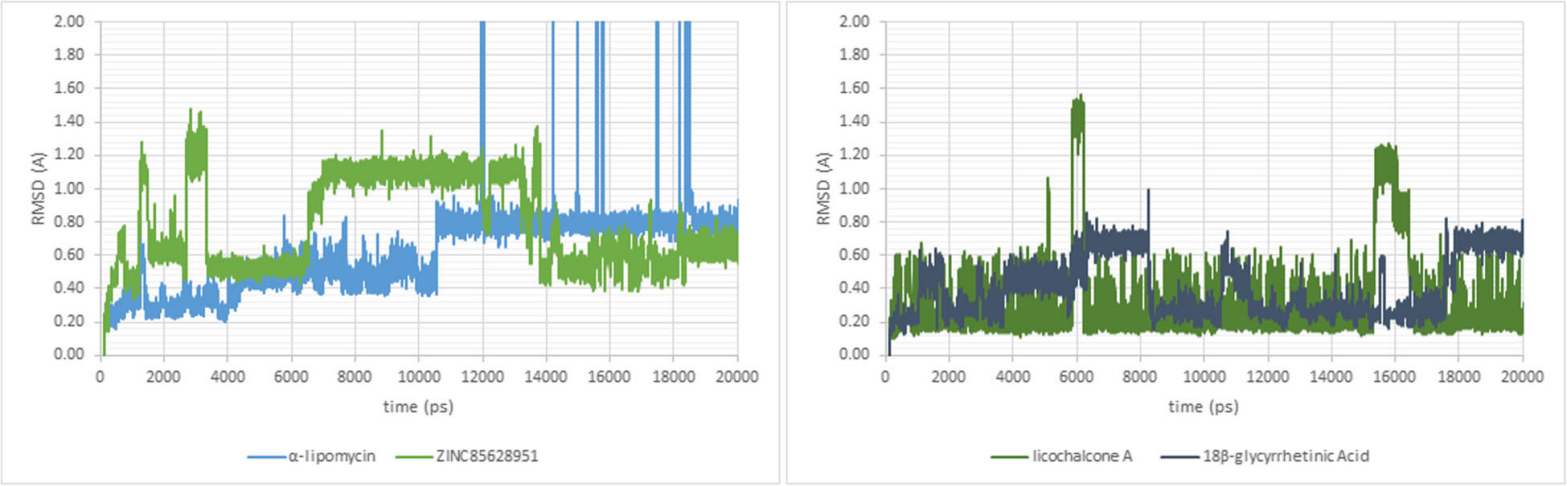
The RMSD curve from molecular dynamics simulation at 20 ns (20.000 ps). The x-axis represents the simulation time (at ps), while the y-axis represents the RMSD value (at nm).

In computational drug discovery, the MD is essential to mimic the conditions of wet experiments, either in vivo or in vitro. This technique could provide the insight about the cryptic or allosteric binding sites of the protein, conformation of ligand-protein complex and could be used to enhancement virtual screening inhibitors methodologies for drug discovery. In the MD simulation, the protein and the ligand could be simulated in condition with varying temperature, time or in any condition that mimics the real-life experiment. It is a beneficial technique to simulate conditions that hard to perform in the wet experiments [62, 63]. In this study, the selected ligands were simulated in MD simulation to determine the stability of the ligand-protein complex when the ligand binds to the protein at its binding site. The simulation comprises of three stages: the first is equilibration stage, this stage was conducted in 100 ps. Also, this stage was also performed to do heating process of the complex, from 300 K to 312 K, to simulate the human body temperature when it infected with Ebola virus and suffered Ebola. After that, a 20,000 ps (20 ns) production stage was performed on the ligand-protein complex to determine its stability. At the end of the simulation, the RMSD value could be observed to predict the ligand-complex complex stability from MD simulation, as it can be seen in Fig. 5. Moreover, the 10 ps-cooling stage was also carried out to see the final interaction in the complex after MD simulation was conducted.

According to Fig. 5, both standard and the best two ligands have retained its binding affinity and still firmly bound to their respective binding site. While the RMSD value that produced in each complex were different; the α-lipomycin complex was stable at 0.80 nm, and ZINC85628951 at 0.60 nm. These results indicate that the complexes that were formed are less stable than the standard ligands (licochalcone A at 0.30 nm and 18β-glycyrrhetinic acid at 0.60 nm, respectively). Moreover, the RMSD graphs of all three best ligands fluctuated before the simulations ended. This is different than EBOV NP-licochalcone A complex, which the complex was more stable because its RMSD value tends to be stable at 0.30 nm, only fluctuated once at 16 ns.

**Fig. 6:**
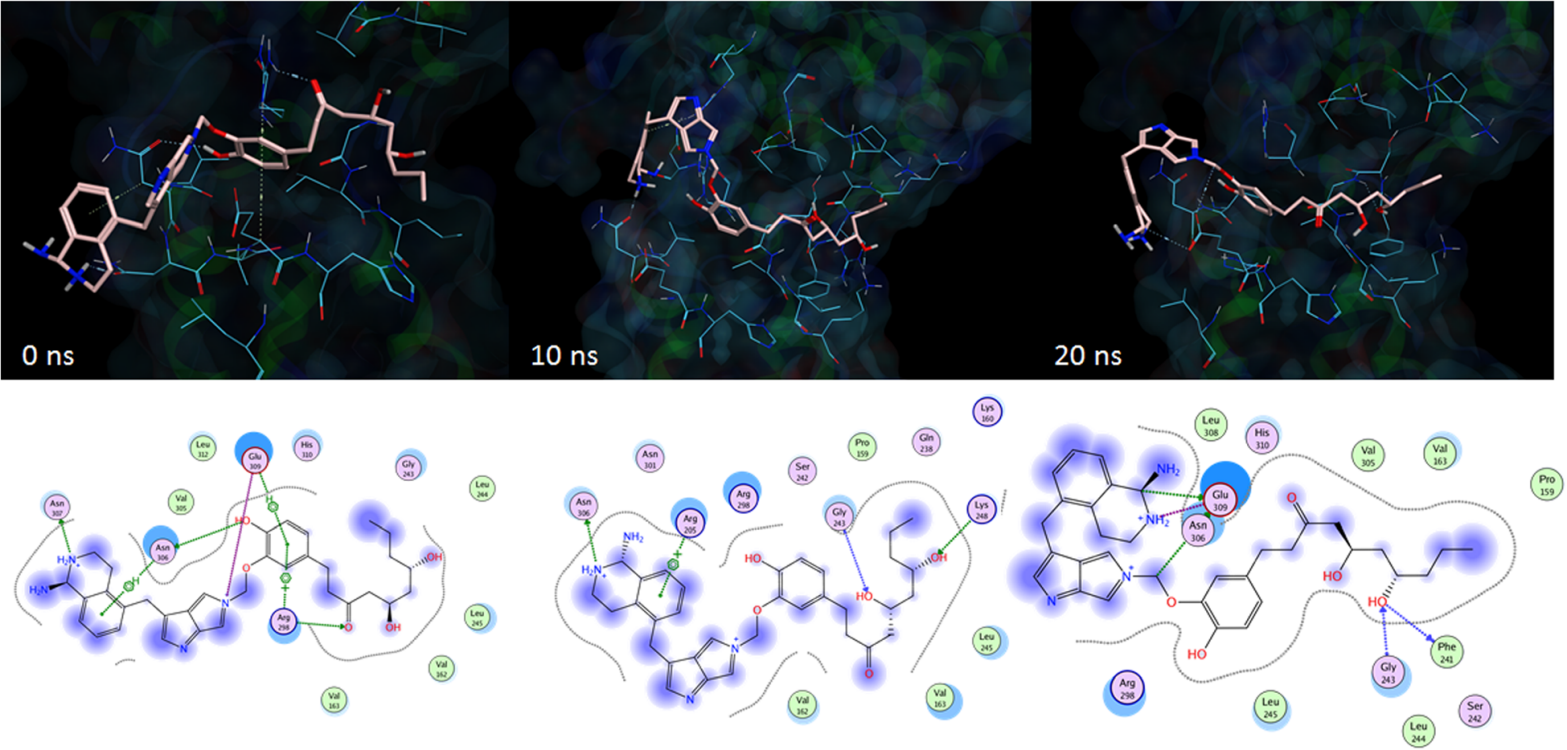
The 3D (top) and 2D (bottom) molecular interaction between EBOV NP and ZINC85628951 at the RNA-binding groove after equilibration process (left), when 10 ns (center), and 20 ns (right) dynamics simulation was occurred

Finally, the molecular interactions of the complex were compared before and after MD simulation was conducted. In this case, EBOV NP-ZINC85628951 complex was chosen for this study. According to Fig. 6, when the EBOV NP-ZINC85628951 complex had entered from equilibration stage into production stage (at 0 ns), the ligand still interacted with Glu309 and Arg298 through pi-pi interaction and hydrogen bond interaction. Moreover, the interaction between Asn307 with the ligand through hydrogen bond interaction was also observed. While the hydrogen bond interaction between His310 with the ligand was vanished. Interestingly, although the Glu309 was still retaining its interaction with ZINC85628951 until the simulation ended, the interaction was briefly lost during the 10 ns dynamics simulation, and the Glu309 interacted through the different site of the ligand. Additionally, at the end of 20 ns dynamics simulation, ZINC85628951 still interacted with two RNA-binding grooves (Arg298 and His310) even though the van der Waals interaction was occurred instead of hydrogen bond interaction (during the docking simulation). Thus, although these ligands have potential to become EBOV NP inhibitor due to their acceptable interactions at the RNA-binding site, even after the MD simulation occurred, more computational studies are recommended to determine the stability of these ligand-protein complexes in longer time simulations.

## Conclusions

Natural product compounds, due to their outstanding bioactivities and unique bioavailability, have been highly regarded as one of the most potent sources of many drugs, with their antiviral activities have been known in recent decades. In this study, about 190,084 natural product compounds from ZINC15 database were obtained to undergo several simulations, including molecular docking simulation, computational ADMET test, and MD simulation. In the end, we discovered that two natural product compounds, namely α-lipomycin (ZINC56874155) and ZINC85628951, were potential to be developed as a novel drug candidate for Ebola, targeting EBOV NP. Thus, we expected that these compounds could be further studied through another computational study and wet lab experiments to prove their inhibition activity and drug potential against EBOV NP.

## Abbreviations

EBOV: Ebola virus
MD: Molecular dynamics
NP: Nucleoprotein
